# Supplementary figures and images for: GSTP1 positive prostatic adenocarcinomas are more common in Black than White men in the United States
Source: PLoS One. 2021 Jun 30;16(6):e0241934. doi: 10.1371/journal.pone.0241934 (PMC8244883; doi:10.1371/journal.pone.0241934)

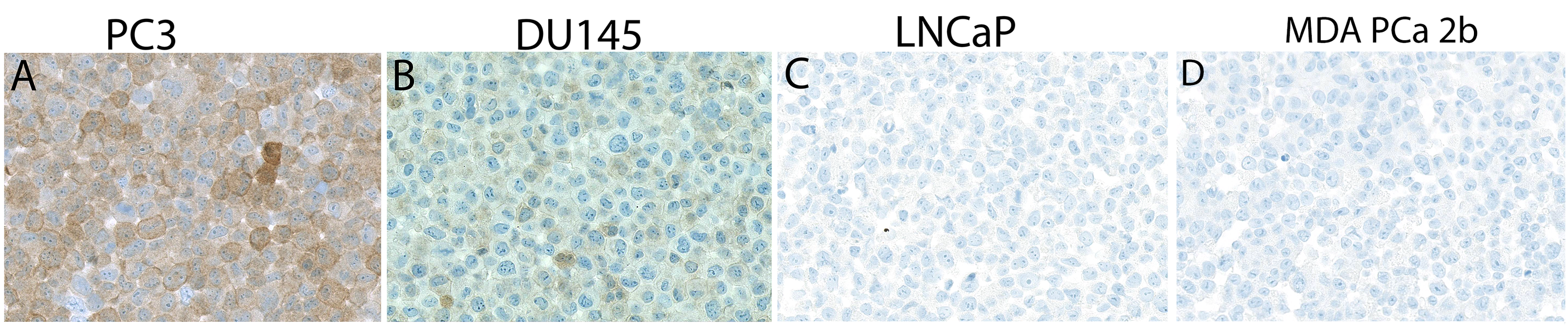

Supplement: S1 Fig — IHC was performed in the cell lines indicated. PC3 and DU145 cells are positive and LNCaP and MDA-PCa-2b are negative. (TIF) [file pone.0241934.s001.tif]
